# The pathophysiology of extracellular hemoglobin associated with enhanced oxidative reactions

**DOI:** 10.3389/fphys.2014.00500

**Published:** 2015-01-14

**Authors:** Joseph M. Rifkind, Joy G. Mohanty, Enika Nagababu

**Affiliations:** ^1^Molecular Dynamics Section, Laboratory of Molecular Gerontology, National Institute on AgingBaltimore, MD, USA; ^2^Department of Anesthesiology and Critical Care Medicine, The Johns Hopkins Medical InstitutionsBaltimore, MD, USA

**Keywords:** extracellular hemoglobin, hemoglobin autoxidation, oxidative reactions, hydrogen peroxide, heme, Fe(IV)hemoglobins, proinflammatory reactions

## Abstract

Hemoglobin (Hb) continuously undergoes autoxidation producing superoxide which dismutates into hydrogen peroxide (H_2_O_2_) and is a potential source for subsequent oxidative reactions. Autoxidation is most pronounced under hypoxic conditions in the microcirculation and for unstable dimers formed at reduced Hb concentrations. In the red blood cell (RBC), oxidative reactions are inhibited by an extensive antioxidant system. For extracellular Hb, whether from hemolysis of RBCs and/or the infusion of Hb-based blood substitutes, the oxidative reactions are not completely neutralized by the available antioxidant system. Un-neutralized H_2_O_2_ oxidizes ferrous and ferric Hbs to Fe(IV)-ferrylHb and OxyferrylHb, respectively. FerrylHb further reacts with H_2_O_2_ producing heme degradation products and free iron. OxyferrylHb, in addition to Fe(IV) contains a free radical that can undergo additional oxidative reactions. Fe(III)Hb produced during Hb autoxidation also readily releases heme, an additional source for oxidative stress. These oxidation products are a potential source for oxidative reactions in the plasma, but to a greater extent when the lower molecular weight Hb dimers are taken up into cells and tissues. Heme and oxyferryl have been shown to have a proinflammatory effect further increasing their potential for oxidative stress. These oxidative reactions contribute to a number of pathological situations including atherosclerosis, kidney malfunction, sickle cell disease, and malaria. The toxic effects of extracellular Hb are of particular concern with hemolytic anemia where there is an increase in hemolysis. Hemolysis is further exacerbated in various diseases and their treatments. Blood transfusions are required whenever there is an appreciable decrease in RBCs due to hemolysis or blood loss. It is, therefore, essential that the transfused blood, whether stored RBCs or the blood obtained by an Autologous Blood Recovery System from the patient, do not further increase extracellular Hb.

## Introduction

The functional role of Red Blood Cells (RBCs) is the transport of oxygen from the lungs to the tissues providing the oxygen required by all cells and tissues. Hemoglobin (Hb) accounts for 95–97% of the cytosolic proteins inside the RBC, and the reversible binding of oxygen to Hb provides the mechanism for oxygen transport by the RBC. Oxygen binds to Hb in the lungs at high partial pressures of oxygen and is released to the tissues at reduced partial pressures of oxygen in the microcirculation. There is, however, a continuous slow autoxidation of Hb when bound to oxygen producing superoxide that converts functional Hb-Fe(II) into oxidized Hb-Fe(III), which no longer binds oxygen (Equation 1).

(1) $$\text{Hb-Fe}(\text{II}){\text{O}}_{2}↔\text{Hb-Fe}(\text{III})+{\text{O}}_{2}^{\cdot -}$$

Hydrogen peroxide (H_2_O_2_) is produced during Hb autoxidation by the spontaneous and enzyme driven dismutation of superoxide (Equation 2).

(2) $$2{\text{O}}_{2}^{\cdot -}+2{\text{H}}^{+}\to {\text{H}}_{2}{\text{O}}_{2}+{\text{O}}_{2}$$

In an intact RBC there are enzymes that reduce oxidized Hb back to functional Fe(II)-Hb. In addition, the reactive oxygen species (ROS), H_2_O_2_ and superoxide, are neutralized by the extensive RBC antioxidant system involving both non-enzymatic low molecular weight antioxidants like glutathione, Vitamin E, and ascorbic acid and enzymatic antioxidants including superoxide dismutase, catalase (Gonzales et al., 1984), glutathione peroxidase (Nagababu et al., 2003), and peroxiredoxin-2 (Lee et al., 2003; Nagababu et al., 2013). Considering the large pool of Hb and the constant slow autoxidation reaction, the intact RBC protects the body from a potential major source of oxidative stress.

The only non-neutralized ROS generated by intact RBCs involves the small fraction of Hb that binds to the RBC membrane. ROS generated near the membrane are less accessible to the cellular antioxidant system, which is primarily located in the cytoplasm. Furthermore, these ROS are being generated where they can damage the RBC membrane and/or be released from the cell to damage other cells and tissues.

The contribution of hemoglobin-membrane interactions is amplified in the microcirculation where Hb is partially oxygenated. Partial oxygenation results in a Hb conformational change that produces a dramatic increase in the rate of autoxidation and the affinity of Hb for the RBC membrane (Cao et al., 2009). The increased autoxidation of cytoplasmic Hb and subsequent generation of ROS can still be neutralized by the cytoplasmic antioxidant system. However, partial oxygenation produces a significant increase in the formation of ROS at the membrane. The potential source for oxidative stress involving membrane binding is, nevertheless, limited by the fact that even under optimal conditions less than 1% of the intracellular Hb can interact with the membrane at any time.

## Extracellular hemoglobin as a source for oxidative stress

Extracellular Hb, whether originating from hemolysis of RBCs or the infusion of cell-free Hb-based blood substitutes, can be a major source for oxidative stress. Under normal conditions this potential source of oxidative stress is minimized by haptoglobin and hemopexin, which bind Hb and free heme, respectively. They inhibit the oxidative reactions of Hb and heme and facilitate their removal from circulation. Elevated levels of free extracellular Hb and heme, which cannot be neutralized by reacting with haptoglobin and hemopexin, have been shown to produce multiple adverse clinical effects.

The mechanisms for these pathophysiological effects have been extensively discussed. A great deal of interest in recent years has been directed at the reaction of NO with Hb (Rother et al., 2005), which reduces the level of NO available for many essential functions. These include regulation of vascular tone, smooth muscle relaxation, neutrophil adhesion to endothelial cells (EC) and platelet activation. The scavenging of NO by extracellular Hb clearly has significant pathological effects.

However, the pathological effects involving oxidative reactions of extracellular Hb also need to be considered.

### Increased autoxidation

**Figure 1:**
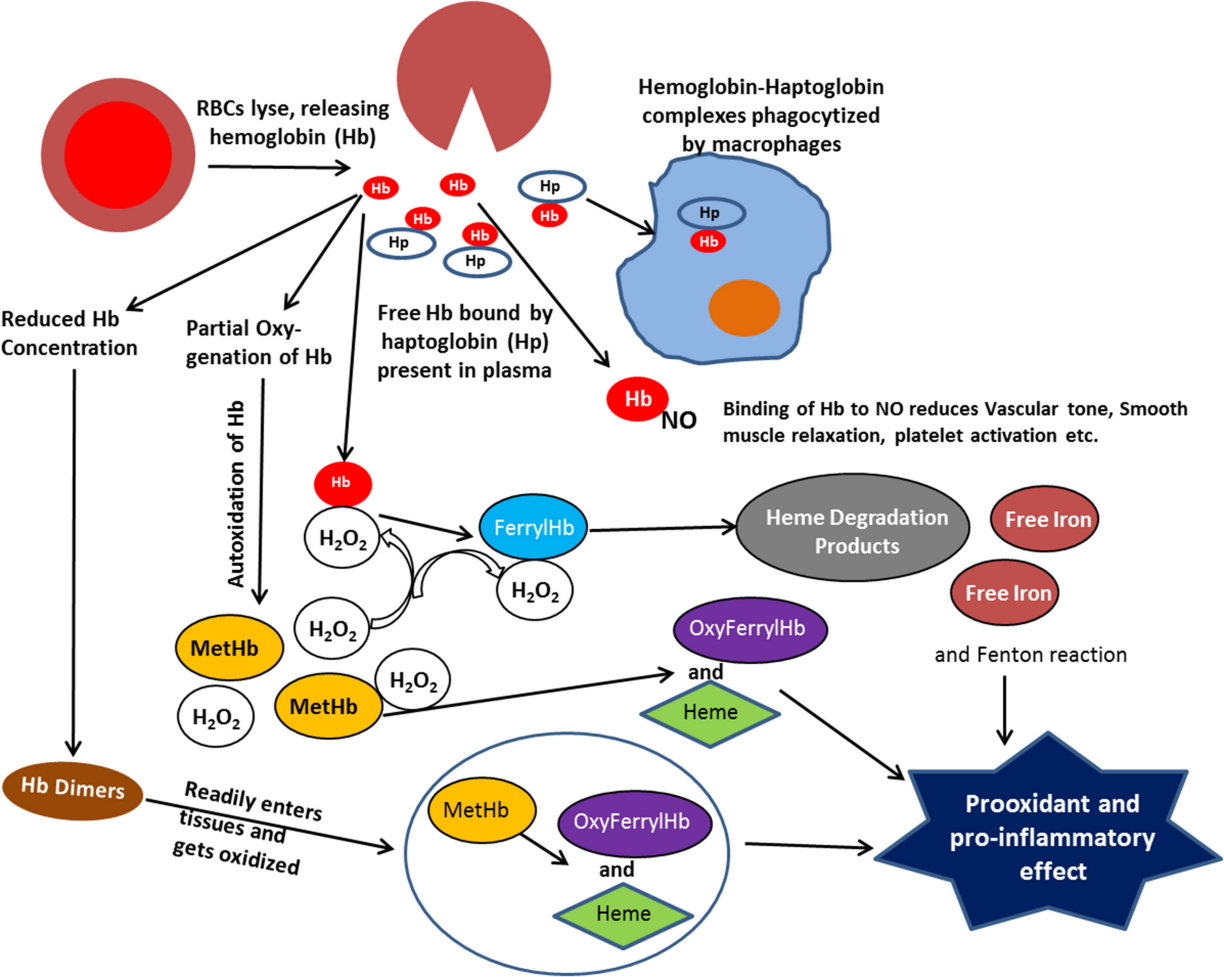
**Scheme illustrating the various reactions that take place with extracellular hemoglobin**.

The oxidative reactions, which occur in RBCs, are greatly amplified at the membrane site where intracellular antioxidant system becomes inefficient. The potential oxidative stress resulting from extracellular Hb is further exacerbated by a dramatic increase in rates of autoxidation for partially oxygenated Hb formed in the microcirculation, as well as an increase in the rate of autoxidation of Hb dimers formed when the Hb tetramer dissociates into dimers (Figure 1) at the reduced Hb concentration in plasma (Zhang et al., 1991). The lower molecular weight of Hb dimers also facilitates the translocation of Hb from the circulation to the vasculature and other tissues sensitive to Hb oxidative reactions (Figure 1).

With extracellular Hb we not only need to deal with the autoxidation reaction and the resultant formation of superoxide and H_2_O_2_, but we need to consider the secondary oxidative reactions involving reactions of H_2_O_2_ with Hb. It has also been shown that a direct reaction of H_2_O_2_ with the iron of Fe(II)-hemoglobin can undergo a Fenton reaction producing the highly reactive hydroxyl radical (Equation 3) (Sadrzadeh et al., 1984).

(3) $${\text{H}}_{2}{\text{O}}_{2}+\text{Fe}(\text{II})\to \cdot \text{OH}+{\text{OH}}^{-}+\text{Fe}(\text{III})$$

The potential significance of this reaction is indicated by the reported detection of hydroxyl radicals in sickle erythrocytes (Hebbel, 1985). Because of their high reactivity any formation of hydroxyl radicals can produce cellular and tissue damage.

### Secondary oxidation reactions involving release of heme and formation of ferryl hemoglobins

The two electron oxidation of Fe(II)-Hb by H_2_O_2_ (Equation 4) produces the Fe(IV)-ferrylHb (Figure 1).

(4) $$\text{Hb}(\text{II})+{\text{H}}_{2}{\text{O}}_{2}\to \text{Hb}(\text{IV})=\text{O}$$

Although the ferrylHb can be a source for oxidative damage, toxic effects due to reactions of ferrylHb have not been demonstrated. However, ferrylHb has been shown to react with an additional molecule of H_2_O_2_ (Equation 5)

(5) $$\begin{array}{l} \text{Hb}(\text{IV})\text{​​}=\text{​​O}+{\text{H}}_{2}{\text{O}}_{2}\to \text{Hb}(\text{III})+{\text{O}}_{2}^{\cdot -}\to \text{heme }\deg\text{radation}\\ \text{ products}+\text{Fe}(\text{III}) \end{array}$$

resulting in the degradation of the heme and the release of fluorescent heme degradation products and free iron (Figure 1) (Nagababu and Rifkind, 1998, 2000). An increase in these heme degradation products has been shown to indicate an increase in oxidative stress (Nagababu et al., 2008a,b), even though specific toxic reactions involving these products have not been established. However, increased Fenton chemistry involving free iron has been well established (Winterbourn, 1995) and the reported increase in free iron for various pathologies, may have originated from this heme degradation reaction (Nagababu and Rifkind, 1998).

The increased formation of superoxide and H_2_O_2_ due to autoxidation of extracellular Hb, also results in the oxidation of the functional ferrous Hb to Fe(III)metHb (Equation 1). Although high concentrations of metHb (hemoglobinuria) are associated with renal dysfunction (Tracz et al., 2007), direct pathological effects of Fe(III)Hb have not been established. However, the heme, which has a lower affinity for metHb than ferrous Hb dissociates from metHb (Bunn and Jandl, 1968) (Figure 1). Heme, a low molecular weight hydrophobic molecule is taken up by cell membranes, plasma proteins, and lipids. Its reaction with low-density lipoproteins has been reported to produce the more toxic oxidized low-density lipoprotein (Balla et al., 1991). Associated with plasma proteins like albumin it is also trans-located to various tissues (Schaer et al., 2013), where it can further generate toxic effects. Heme can bind to certain receptors, transcription factors, and enzymes. These interactions can alter cellular function, metabolism, and gene transcription. The altered gene transcription is the basis for a heme induced proinflammatory effect (Figure 1) (Wagener et al., 2001b).

The two electron oxidation of Fe(III)Hb by H_2_O_2_ produces oxyferrylHb

(6) $$\text{Hb}(\text{III})+{\text{H}}_{2}{\text{O}}_{2}\to \cdot \text{Hb}(\text{IV})=\text{O}+{\text{H}}_{2}\text{O}$$

The oxyferrylHb is unstable with the unpaired electron migrating to the globin with the formation of radicals involving tyr-24, tyr-42, and his-20 of the alpha chain and tyr-35, tyr-130, and cys-93 of the beta chain (Deterding et al., 2004). These products are highly reactive and have been shown to produce crosslinked Hb multimers (Nagy et al., 2010). OxyferrylHb, like metHb, has been shown to release heme. However, oxyferryl and/or the resultant multimers without dissociating heme act as a proinflammatory agonist attacking the vascular endothelium (Silva et al., 2009).

Both heme and oxyferrylHb have been reported to be proinflammatory agonists (Figure 1) resulting in the activation of the redox sensitive transcription factor NF-κB. However, two different pathways are involved. The proinflammatory effect of oxyferrylHb is triggered by interactions of multimeric hydrophobic aggregates recognized by innate receptors (Silva et al., 2009). This disrupts membranes and triggers inflammation via an IL-1/1L-1Ra signal transduction pathway. Heme, however, can bind to TLR4 (Teng et al., 2009). The interaction with TLR4 induces activation of NF-κB (Lin et al., 2012).

## Pathological effects associated with hemoglobin oxidative reactions

In attempting to evaluate the pathological effects of oxidative reactions involving extracellular Hb, the first area to look at are effects on the plasma lipoproteins and the vasculature, which Hb comes directly in contact with.

### Lipoproteins and the vasculature

Oxidation of low density lipoproteins (LDL) is thought to play an important role in initiating atherogenesis (Steinbrecher et al., 1984). The oxidation of LDL is triggered by the uptake of heme by LDL. It has been reported that the LDL, which is highly hydrophobic, is able to compete with hemopexin and albumin for the free heme, with 80% of heme added to plasma immediately taken up by lipoproteins (Miller and Shaklai, 1999). The LDL oxidative reactions involve low levels of lipid hydroperoxides or oxidants, which lead to oxidative degradation of the heme and the release of heme iron (Sadrzadeh and Eaton, 1988). It is thought that it is this free iron that results in the accelerated oxidation of polyunsaturated fatty acids and other components of the LDL.

The atherosclerotic plaques and particularly disruptured plaques have been shown (Nagy et al., 2010) to lyse RBCs and oxidize the released Hb producing metHb, free heme, free iron and the Fe(IV)Hb oxidized product, oxyferrylHb. Dityrosine and cross-linked hemoglobins produced as a result of the formation of oxyferrylHb are also detected. In addition to the heme released from metHb, it has been shown that heme is released from oxyferrylHb. The enhanced release of heme and free iron due to the interaction of plaque with RBCs further amplify the oxidation of plaque components that results in cytotoxic reactions affecting the endothelium (Nagy et al., 2010).

The oxidative reactions discussed are thought to primarily involve the release of heme and iron. However, *in vitro* studies have demonstrated that oxyferrylHb also acts as a proinflammatory agonist (Silva et al., 2009). *In vitro* studies have shown that oxyferrylHb induces the formation of F-actin stress fibers resulting in the formation of intercellular gaps disrupting the integrity of the endothelium. This results in extravascular leakage. This leakage activates the κB family of transcription factors inducing the expression of proinflammatory genes like E-selectin, ICAM-1, and VCAM-1. In addition, oxyferrylHb activates the signal transduction pathways involving p38 MAPK and JNK. This proinflammatory response was shown to increase EC permeability and enhance monocyte adhesion. The combined oxidative effects due to both metHb, oxyferrylHb, and free heme increase oxidation of plaque lipids, while the proinflammatory effects of oxyferrylHb trigger endothelial cytotoxicity. Together, these processes play a significant role in the pathology associated with atherosclerosis.

### Renal dysfunction

In addition to the oxidative reactions involving lipoproteins and the vasculature, renal dysfunction is a major pathology that results from oxidative reactions associated with extracellular Hb. This pathology is triggered by the uptake of Hb dimers by the kidney glomerulus. Hb dimers are formed from extracellular Hb due to the dissociation of tetrameric Hb into dimers (Ackers and Halvorson, 1974) at the reduced Hb concentration in plasma. The reduced molecular weight of the Hb dimers (32-kD instead of 64-kD) facilitates their transfer into tissues. However, the uptake of Hb dimers is most pronounced in the kidney that is designed to remove free Hb from the circulation.

As discussed above, the extensive RBC antioxidant system severely limits oxidative reactions involving intracellular Hb. The antioxidant capacity of plasma is appreciably less relative to that of the RBCs. Nevertheless, the reducing ability of ascorbic acid and urate in plasma results in relatively low levels of oxidized Hb in plasma (Butt et al., 2010). Hb dimers translocated into the kidney, experience a much harsher oxidative environment as indicated by the high levels of metHb in the urine when elevated cell free Hb is present (Boretti et al., 2009). The increased Hb oxidation in the kidney also results in the subsequent release of free heme. Free heme in the kidney as well as other organs induce heme oxygenase-1, which converts heme to bilirubin with antioxidant activities (Stocker et al., 1987). Excess heme in the kidney as well as other organs and tissues produce a number of cytotoxic effects.

The hydrophobic heme in cellular membranes can oxidize lipids, denature proteins, and perturb the integrity of the attached cytoskeleton. Heme can oxidatively denature DNA and impair the activity of cytosolic enzymes including glucose-6-phosphate dehydrogenase and glutathione reductase. Heme can also activate cell-damaging enzymes such as caspases and cathepsins (Tracz et al., 2007). Heme affects mitochondrial function with an initial increase in respiration followed by a decrease and ultimate cessation of oxygen consumption (Nath et al., 1998). Even relatively low levels of heme become cytotoxic in the presence of H_2_O_2_, which can degrade the heme producing free iron.

In addition to the direct oxidative reactions involving the heme, elevated heme levels have also been shown to have a proinflammatory effect (see above). Studies involving the vasculature (Silva et al., 2009) suggest that metHb does not have a proinflammatory effect and that reported effects due to metHb (Liu and Spolarics, 2003; Silva et al., 2009) can be due to endotoxin contamination. There is, however, clear evidence that heme, which readily dissociates from metHb, does have a proinflammatory effect even in the absence of endotoxin (Fortes et al., 2012).

The heme induced proinflammatory effect in the kidney induces the chemokine monocyte chemo-attractant protein-1 (MCP-1) and transforming growth factor b1 isoform1 (TGF-beta-1) by activating the redox sensitive transcription factor NF-κB (Kanakiriya et al., 2003). The resultant increased levels of the chemokines and other proinflammatory reactions, are thought to contribute to tubule-interstitial disease (Qian et al., 2010), decreased renal perfusion and intra-tubular casts formed by the interaction of heme proteins with Tamm-Horsfall protein (Tracz et al., 2007). Pathology of Tamm-Horsfall protein (Hoyer and Seiler, 1979) can result in chronic renal dysfunction.

### Other toxic effects due to hemoglobin oxidative reactions

While these effects are generally most pronounced in the kidney, which plays a major role on removing the extracellular Hb from the body, similar affects are observed in other tissues where Hb is translocated from the plasma into tissues and exposed to increased oxidative conditions. Proinflammatory effects of free heme have been shown to induce up-regulation of adherence molecules in other organs including the gut, liver, and pancreas (Wijayanti et al., 2004). This increased adherence results in leukocyte recruitment and increased vascular permeability (Wagener et al., 2001b). These effects are particularly relevant in diseased and inflamed tissues or after an ischemic insult when other oxidants are present in the tissue. Heme proinflammatory effects have also been shown to induce programmed necrosis on macrophages (Fortes et al., 2012) and to be involved in intracerebral hemorrhage (Simoes et al., 2013). In sickle cell disease, it has been shown that heme released into the circulation causes expression of endothelial adhesion molecules, which results in increased adhesion of leukocytes and reticulocytes to the endothelium (Wagener et al., 2001a). It has also been shown that free heme precipitates severe malaria and particularly cerebral malaria (Ferreira et al., 2008). This is associated with the disruption of the blood brain barrier and adhesion of infected RBCs to the brain microvascular endothelium. These processes are thought to involve ROS, but the adherence could involve the heme induced inflammatory response. In conclusion, the combined effects of intrinsic oxidative reactions and oxidative reactions triggered as a result of inflammation have an important role in the pathology that results from increased concentrations of extracellular Hb.

### Physiological sources for cell free hemoglobin

Under normal conditions minimal lysis of RBCs occur before the aged cells are removed from circulation. The level of cell free Hb in circulation is, therefore, minimal. Increased levels of extracellular Hb occur when the rate of RBC lysis increases and the removal of extracellular Hb from circulation are slower than the production of additional Hb from cell lysis.

Increased RBC lysis *in vivo* occurs due to one of two classes of pathological situations.

(1) Modifications of the RBC that decrease its stability and increase lysis as the cells flow through the circulatory system. These changes generally involve alteration of the genes that control RBC production and function. This can affect the Hb, the RBC membrane or enzymes required for proper function of the RBC. For example, in Sickle Cell Anemia and Thalassemia the altered Hbs are less stable resulting in increased intracellular oxidative reactions that damage the cell membrane and increase RBC lysis (Nagababu et al., 2008a). Sickle cell anemia involves a mutation on the B6 residue that results in a tendency for the hemoglobin to aggregate resulting in an appreciable shorter cell lifetime (Wagener et al., 2001a). In Thalassemia, adequate levels of the alpha chain or beta chain are not produced and thus there is disruption of the stable quaternary structure of Hb (Kidd et al., 2001), resulting in impaired stability and increased RBC lysis.

Examples of altered membrane structure are Hereditary Spherocytosis and Hereditary Elliptocytosis, in which the shape of the RBC is spherical and elliptical, respectively, instead of the normal biconcave shaped cells (Da et al., 2013). The altered cells are less deformable and have difficulty passing through the narrow capillaries in the microcirculation resulting in increased cell lysis. Paroxysmal Nocturnal Hemoglobinuria also results in altered RBC membrane structure (Basu et al., 2010) that results in increased clots in the veins.

Examples of altered enzyme activity that affect the ability of RBCs to withstand oxidative stress are glucose-6-phosphate dehydrogenase (G6PD) deficiency (Rochford et al., 2013) and pyruvate kinase deficiency (Abdel et al., 2010). G6PD plays an essential role in the phosphate shunt that helps maintain the RBC antioxidant activity and pyruvate kinase is an essential enzyme involved in glucose metabolism and the production of ATP. The resultant altered metabolic activity results in increased oxidative stress and increased cell lysis.

*In vitro* or *in vivo* processes that stress the RBC membranes contribute to mechanical hemolytic anemia. This process is analogous to the increased lysis that occurs in certain abnormal Hbs with altered cell shape (see above) and with sickle cell disease where the sickling produces a decrease in deformability. A number of treatments used also mechanically stress the RBC membrane and can induce increased lysis. This includes an artificial heart valve, hemodialysis for kidney failure, heart lung bypass machine used for open-heart surgery, preeclampsia, and malignant hypertension.

(2) Situations where the RBCs may be normal but diseases cause the RBCs to be lysed. Autoimmune Hemolytic Anemia (AIHA) is a situation where the immune system makes antibodies that attack the RBC (Wakui et al., 1988). Although what triggers AIHA is not known, certain diseases or infections involving inflammation such as lupus, hepatitis, and HIV increase the risk for AIHA. Alloimmune hemolytic anemia (Odabas et al., 2002) involves antibodies made against blood obtained from a blood transfusion, if the transfused blood is of a different type than that of the patient. Drug-induced hemolytic anemia is induced by certain medicines such as those used in chemotherapy or medicines used to treat malaria. Many disease situations that result in increased oxidative reactions/inflammation in specific tissues can cause lysis of the RBCs when the RBCs come in contact with these inflamed tissues. For example, RBC lysis is induced when they come in contact with atherosclerotic tissue (see above).

The increased extracellular Hb resulting from hemolytic anemia is amplified by the frequent infusion of blood to compensate for the reduced RBC content and the resultant impaired delivery of oxygen. The required infusion of blood is most pronounced during surgeries that involve an appreciable loss of blood.

Initially prompted by the need for a source of blood available in the battlefield where whole blood is not available, a number of studies focused on the use of Hb based blood substitutes. The Hb in these blood substitutes were modified to adjust the oxygen affinity to that of whole blood and cross-linked forming higher molecular aggregates to prevent the rapid removal of cell free Hb by the kidneys. The initial failure to consider the difficulties associated with cell-free Hb involving both NO complexation and oxidative reactions resulted in very limited use of Hb based blood substitutes until modification can be put in place to minimize these serious side effects.

The two sources of blood that is currently in use involve (1) stored whole blood collected from volunteers and (2) RBCs obtained from an Autologous Blood Recovery System like “Cell Saver” (Ottesen and Froysaker, 1982). Cell Saver blood is collected from patients during surgeries involving a major loss of blood. Since this Cell Saver blood has anticoagulant added, cells are washed to remove excess anticoagulant, plasma, platelets, leukocytes, and any free Hb. The washed and packed RBCs are then suspended in saline to a 50% hematocrit and transferred to a transfer pack for reinfusion when needed. If necessary, the reinfusion can occur less than 10 min after the collection of the blood (Keeling et al., 1983).

Blood, even *in vivo*, has a limited lifespan of 120 days. After removal from the circulation there are metabolic and oxidative changes that begin to take place immediately. To minimize these effects blood is stored in a media that is supposed to minimize these effects. Currently the FDA permits the use of stored blood for 42 days. Recent studies, however, indicate increased oxidative reactions as well as instability of the RBC membrane resulting in increased rates of lysis and more rapid removal from circulation well before 42 days storage (Wang et al., 2012).

Cell Saver RBCs are from patient's fresh blood and therefore minimizes the major metabolic and oxidative difficulties associated with stored blood. However, recent studies (Gueye et al., 2010) indicate that these cells are, nevertheless, susceptible to lysis and the production of extracellular Hb. Some of the reported increase in lysis can be related to the fact that we are using blood of patients with various diseases, which presumably involve increased inflammation and oxidative stress.

Considering the major difficulties associated with extracellular Hb, additional studies are crucial. For stored blood methods to extend the stability of the stored RBCs are essential. For Cell Saver blood cells where we are dealing with fresh blood it is essential to identify the source for any increased lysis and develop methods to minimize lysis and the formation of extracellular Hb.

### Conflict of interest statement

The authors declare that the research was conducted in the absence of any commercial or financial relationships that could be construed as a potential conflict of interest.
